# Management of good-risk metastatic nonseminomatous germ cell tumors of the testis: Current concepts and controversies

**DOI:** 10.4103/0970-1591.60449

**Published:** 2010

**Authors:** Gautam Jayram, Russell Z. Szmulewitz, Scott E. Eggener

**Affiliations:** Section of Urology, Department of Surgery, University of Chicago Medical Center, USA; 1 Department of Medicine, University of Chicago Medical Center, USA

**Keywords:** Metastatic germ cell cancer, non-seminoma, RPLND, testicular cancer, testis

## Abstract

**Introduction/Methods:**

Approximately 30% of nonseminomatous germ-cell tumors (NSGCT) of the testis present with metastatic disease. In 1997, the International Germ Cell Cancer Collaborative Group (IGCCCG) stratified all patients with metastatic NSGCT into various risk groups based on serum tumor markers and presence of visceral disease. We review the literature and present optimal stage-dependent management strategies in patients with favorable-risk metastatic NSGCT.

**Results:**

Primary chemotherapy (3 cycles BEP or 4 cycles EP) has been shown to be the preferred modality in patients with Clinical Stage IS (cIS) and in patients with bulky metastatic disease (≥CS IIb) due to their high risk of systemic disease and recurrence. Primary retroperitoneal lymph node dissection appears to be the most efficient primary therapy for retroperitoneal disease <2 cm (CS IIa), with adjuvant chemotherapy reserved for patients who are pathologically advanced (>5 nodes involved, single node > 2 cm) and for those who are non-compliant with surveillance regimens. Following primary chemotherapy, STM and radiographic evaluation are used to assess treatment response. For patients with normalization of STM and retroperitoneal masses < 1 cm, retroperitoneal lymph node dissection or observation with treatment at disease progression are considered options. Due to risk of teratoma or chemoresistant GCT, masses >1 cm and extra-retroperitoneal masses should be treated with surgical resection, which should be performed with nerve-sparing, if possible.

**Conclusions:**

In patients with favorable disease based on IGCCCG criteria, clinical stage, STM, and radiographic evaluation are used to guide appropriate therapy to provide excellent long-term cure rates (>92%) in patients with metastatic NSGCT.

## INTRODUCTION

Since the development and routine use of cisplatin- based chemotherapy regimens in the 1970s, the cure rate for testicular cancer has increased substantially even in the advanced, metastatic setting.[1,2] Apart from its potential lethality, testicular cancer remains a significant cause of short and long-term morbidity, which is particularly concerning in an otherwise healthy cohort of young men with prolonged life expectancies. With the standardization of patient risk into good, intermediate, and poor-risk groups at the time of diagnosis (IGCCCG classification), clinicians can more accurately estimate prognosis and tailor therapy [Table 1].[2] Chemotherapy remains the mainstay of primary therapy for intermediate and high-risk non-seminomatous germ cell tumors (NSGCT).

**Table 1:** IGCCCG risk stratification of NSGCT

Good-Risk
No primary mediastinal tumor
Absence of non-pulmonary visceral metastases
Favorable serum tumor markers:
AFP < 1,000 ng/ml
hCG < 5,000 IU/ml
LDH < 1.5×Normal limit
Intermediate-Risk
No primary mediastinal tumor
Absence of non-pulmonary visceral metastases
Elevated serum tumor markers:
AFP 1,000 ng/ml – 10,000 ng/ml
hCG 5,000 IU/ml – 50,000 IU/ml
LDH 1.5 -10×Normal limit
Poor-Risk (Presence of any of the following):
Mediastinal primary tumor
Presence of non-pulmonary visceral metastases
Unfavorable serum tumor markers:
AFP >10,000 ng/ml
hCG > 50,000 IU/ml
LDH > 10×Normal limit

Patients with good-risk metastatic NSGCT have a five-year cancer-specific survival of 92% and may include patients from clinical stage (CS) IS to IIIA.[3,4] Achieving optimal outcomes in this group requires a detailed understanding of metastatic patterns, histologic subtypes, and treatment paradigms. We review the evidence-based management of patients with good-risk metastatic NSGCT.

## CLINICAL STAGE IS NON-SEMINOMATOUS GERM CELL TUMORS

Following radical orchiectomy, patients whose serum tumor markers (STM) fail to normalize in the absence of radiographic disease, regardless of the extent of the primary tumor, are classified as clinical stage IS (cIS). Initial reports studying the efficacy of primary RPLND (p-RPLND) in these patients showed high rates of systemic relapse, persistent marker elevation, and high incidence of pathologic retroperitoneal disease.[5,6] Reported treatment failure rates of 37-100% in these studies strengthened the notion of systemic micrometastatic disease in cIS patients, thus establishing primary chemotherapy as the standard of care [Table 2]. In this setting, induction chemotherapy and surveillance have yielded success rates of 71-75%, with recurrent disease identified by persistent marker elevation or abdominal imaging.[5] Dash *et al*. recently reported on the outcomes of 24 cIS patients and found that in seven patients undergoing post-chemotherapy RPLND (PC-RPLND) (four for recurrence, three electively), six (86%) had chemo-resistant teratoma. The authors concluded that PC-RPLND may benefit select cIS patients with a radiographically normal retroperitoneum.[7] Due to concerns of overtreatment and unnecessary exposure to chemotherapy, Williams *et al*. recently reviewed their experience with p-RPLND in 24 cIS patients.[8] Among 24 patients, 15 (63%) had negative pathologic LN, and of 9 (37%) patients with positive LN, 5 (55%) received adjuvant chemotherapy (3 for recurrence, 2 electively). With a mean follow-up of 2.9 years, all 24 patients are without evidence of disease. Based on this, the authors conclude p-RPLND can potentially spare chemotherapy in this high-risk group, adequately control the retroperitoneum, and allow for administration of adjuvant therapy safely in the setting of recurrence. This represents a departure from the accepted management strategy of primary chemotherapy in cIS and requires further evaluation.

**Table 2:** Primary treatment options for good-risk NSGCT by clinical stage

Stage	Recommended Therapy
IS	Chemotherapy: BEP × 3 or EP × 4
IIA	RPLND + adjuvant chemotherapy*, if needed
IIB – IIIA	BEP × 3 or EP × 4followed by PC-RPLND, if needed

* 2 cycles EP or BEP

## CLINICAL STAGE IIA NSGCT: RETROPERITONEAL LYMPH NODE DISSECTION (RPLND)

Patients with good-risk metastatic NSGCT, as defined by the IGCCCG [Table 1], were all treated initially with chemotherapy. However, patients with normal STM and abdominal imaging demonstrating retroperitoneal lymphadenopathy ≤ 2 cm (CS IIA) are typically treated with primary retroperitoneal lymph node dissection [Table 2], with 60% found to have pathologic stage (PS) II disease.[8] The rationale for primary surgery is based on the retroperitoneum being the first site of extratesticular spread in ^∼^90% of patients and the most frequent site of chemoresistant GCT and teratoma.[9–11] This approach minimizes the need for chemotherapy and its attendant long-term risks, in addition to decreasing cumulative radiation exposure by eliminating the need for routine postoperative abdominal imaging. CT scans are the mainstay of extra-testicular imaging but lead to understaging in approximately 25-30% of all CS I patients. [8] Therefore, RPLND serves a diagnostic, prognostic and therapeutic role in this setting. Cancer recurrence after an appropriately performed RPLND ranges from 1-7%, occurs predominantly in the chest, and is usually easily managed with chemotherapy.[12] Furthermore, increasing awareness of long-term treatment-related morbidity of chemotherapy [Table 3] emphasizes the importance of primary RPLND as a curative option that often obviates the need for systemic therapy. A review of more than 30,000 testicular cancer patients found a 6% higher non-cancer mortality rate in cancer survivors following treatment, compared to matched controls, one year following diagnosis.[13] In addition, a meta-analysis of secondary malignancies in testicular cancer patients estimated 40-year cumulative risks of 31-36% vs.23% for the general population, largely due to the impact of radiotherapy (RR = 2.0) and chemotherapy (RR = 1.8), with combination therapy having the most pronounced impact (RR = 2.9).[14]

**Table 3:** Potential toxicity of chemotherapeutic agents used in NSGCT

Agent	Acute toxicity	Chronic toxicity
Bleomycin	Interstitial pneumonitis/pulmonary fibrosis, skin changes	Decreased FEV1, FVC
Etoposide	Alopecia, cytopenias, nausea/vomiting	Secondary leukemia
Cisplatin	Nausea/vomiting, cytopenias, renal insufficiency,neuropathy, ototoxicity	Nephrotoxicity, ototoxicity, cardiovascular events, azospermia

A cost-benefit analysis of primary chemotherapy versus primary RPLND for patients with CS II NSGCT concluded that survival and quality of life were similar but patients undergoing primary RPLND had less toxicity, lower risk of late relapse, improved fertility, and a favorable cost burden 5 years after treatment.[15] Two separate non- randomized studies have compared primary chemotherapy versus RPLND for patients with CS IIA – IIB NSGCT. Weissbach *et al*. evaluated 187 patients who underwent primary chemotherapy or RPLND plus 2 cycles of adjuvant chemotherapy (if PS II) in a multicenter European trial. Of patients treated with primary RPLND, 12% were PS I, precluding the need for further chemotherapy. Of those receiving primary chemotherapy, 33% required PC-RPLND due to residual or recurrent disease. Overall disease-specific survival was 99% without differences between the two treatment groups and quality-of-life was similar.[16]

The authors favor primary RPLND due to the potential to spare some patients chemotherapy and for those that require adjuvant chemotherapy based on RPLND findings, a less toxic regimen of two cycles compared to the three or four cycles used for primary treatment. A more recent comparison by Stephenson et al. showed superior recurrence-free survival (98% vs.79%) with primary chemotherapy followed by RPLND compared to RPLND alone, with no difference in cancer-specific survival.[12] Given uniformly high primary and salvage cure rate in patients with low-volume disease, patient selection factors become critical to assess those most likely to benefit from primary chemotherapy. Stephenson *et al*. assessed the impact of selection criteria on pathologic findings and relapse rates.[17] The group analyzed 453 patients undergoing primary RPLND from 1989-2002, 32% of whom were CS IIA-IIB, and found 60% of CS IIA patients and 100% of IIB patients with metastases in retroperitoneal lymph nodes. CS IIB and elevated STM were the only pre-RPLND predictors of progression. Based on these findings, there is strong support for RPLND as preferred primary therapy in patients with CS I – IIA disease with normal STM following orchiectomy and reserving primary chemotherapy followed by RPLND for patients with CS IIB or elevated STM.

## CHEMOTHERAPY FOLLOWING PRIMARY RPLND

The risk of recurrence following RPLND depends on nodal status, with pathologic N1 and N2 patients having an estimated risk of recurrence of 8–32% and 50-70%, respectively. Several studies have demonstrated a significant reduction in this risk with immediate chemotherapy for pathologic stage II disease.[17–20] Observation with salvage chemotherapy given at the time of relapse appears to produce similar cure rates compared to immediate adjuvant therapy; however the chemotherapeutic burden and toxicity is higher (three to four cycles versus two cycles). Williams and Einhorn first reported a recurrence rate of 48% in patients with positive nodes (N1 and N2) following RPLND who were observed compared with 2% recurrence in patients given two cycles of adjuvant chemotherapy.[19]

Further evidence supporting this paradigm was an evaluation of 50 patients with pathologic N2 – N3 following RPLND who were given two cycles of adjuvant etoposide and cisplatin.[20] With a median follow-up of 35 months and 84% of patients with greater than two years follow-up, all were alive and free of relapse. Alternatively, observation of low-volume metastatic disease is reasonable as Richie *et al*. followed a group of 39 N1 patients after RPLND and reported a relapse rate of only 8% with a median follow-up of 3.5 years[21] and Stephenson *et al*. reported contemporary N1 patients having a four-year relapse rate of 10% without adjuvant chemotherapy.[17] Based on this data, for compliant patients with completely resected retroperitoneal metastases, observation with salvage chemotherapy at relapse is commonly recommended for patients with pathologic N1 disease whereas two cycles of adjuvant chemotherapy is administered to noncompliant patients or those with pathologic N2 or N3 disease.

## CLINICAL STAGE IIB-III NSGCT: CHEMOTHERAPY

Since the contributions of Einhorn and Donahue in 1977, platinum-based chemotherapy regimens have been standard therapy for patients with advanced disease.[1] Through a series of well-designed clinical trials, two chemotherapy regimens have been established as standards of care: BEP X 3 cycles or EP X 4 cycles [Table 4]. The Indiana University group showed that BEP X 3 was as efficacious as BEP X 4 and associated with less toxicity, shorter duration, and less cost for minimal or moderate risk patients according to their risk criteria, establishing BEP X 3 as a standard option for the IGCCCG good-risk NSGCT population.[22] The initial trials for advanced germ cell tumors used cisplatin combined with vinblastine and bleomycin, which were already established as active agents in testicular cancer.[1,23] However, etoposide soon emerged as an active agent and supplanted vinblastine due to its similar efficacy and improved toxicity profile.[19] A long-term analysis of two randomized trials at Memorial Sloan-Kettering of good-risk patients receiving EP×4 showed a 91% complete response rate and 86% were alive at a median of 7.6 years follow-up.[24]

**Table 4:** Standard primary chemotherapy regimens for good risk NSGCT

**Regimen**	**Drug, dose, schedule**
BEP	Bleomycin 30 units (days 2, 9, 16)
3 cycles, 21 day cycle	Etoposide 100mg/m^2^(days 1-5)
	Cisplatin 20mg/m^2^ (day 1-5)
EP	Etoposide 100mg/m^2^ (days 1-5)
4 cycles, 21 day cycle	Cisplatin 20mg/m^2^ (day 1-5)

Based on these trials, BEP × 3 and EP × 4 have been established as routine regimens for patients with good-risk disease requiring induction chemotherapy. There is little evidence to discriminate which of these two is optimal.[25] An EORTC trial conducted by de Wit *et al*. compared these two regimens and found a superior complete response rate (95% vs. 87%) for the BEP group, however no significant differences in survival or time to progression were observed. [26] More importantly, the BEP group suffered a 1% mortality rate from bleomycin-induced pulmonary toxicity and a significantly higher rate of Raynaud phenomenon. However, this trial must be interpreted with consideration of the etoposide dose used (360/mg/m^2^/cycle), lower than the currently established and more efficacious dose (500/mg/m^2^/ cycle). Culine *et al*. have provided the most recent data using conventional doses of all agents in 270 patients randomized to EP × 4 or BEP × 3.[27] The authors found 97% of the EP group and 95% of the BEP group achieved a complete or partial response. Grade 2 or higher dermatologic (7% vs. 2%) and neurologic side effects (16% vs. 3%) were more frequent in the BEP group without major differences in pulmonary toxicity (4% vs. 2%), although routine pulmonary function testing was not employed. The overall impact of these trials is insufficient to support one regimen over the other, especially from an efficacy standpoint. Less cumulative exposure to bleomycin and reduced morbidity are the most commonly cited rationale by advocates of the EP regimen.

## POST-CHEMOTHERAPY RESIDUAL MASSES

Treatment response following chemotherapy is assessed using STM and radiographic imaging. Virtually all groups agree that residual masses larger than 1-2 cm should be resected but considerable variation exists for complete responses to chemotherapy or residual masses < 2 cm. The management of patients with a post-chemotherapy complete response or small residual mass in the retroperitoneum (particularly less than 1 cm) is controversial. The persistence of elevated STM after initial induction chemotherapy remains the only generally accepted contraindication to immediate post-chemotherapy surgery. Traditionally, residual masses after induction therapy are comprised of teratoma (^∼^40%), necrosis or fibrosis (^∼^40%), or viable chemoresistant GCT (^∼^20%).[28] As chemotherapy regimens have evolved, rates of post-chemotherapy viable GCT have decreased to as low as 11-13%.[29,30] To more accurately select patients for post-chemotherapy surgery, multiple groups have attempted to develop validated models or predictors of teratoma and viable GCT in residual masses. In a group of 556 patients studied by Steyerberg et al, predictors of necrosis were the absence of teratoma in the orchiectomy specimen, normalization of STM, and >90% reduction in tumor size following chemotherapy. [30] Although each of these parameters were associated with post-chemotherapy RP histology, the accuracy for predicting necrosis or fibrosis was 84%, thereby rendering a significant minority of 16% with an inappropriate designation.

Oldenburg *et al*. evaluated patients with < 2 cm residual masses and found a 33% rate of either teratoma or viable GCT in 87 patients. [31] Further, five of six patients with viable GCT had lesions ≤ 1.0 cm. The degree of shrinkage of the mass during therapy or the post-treatment size of the mass did not predict final histology, highlighting the significant limitations of observing all patients with small residual masses. Based on this data, they recommend routine RPLND in the setting of small post-chemotherapy masses as opposed to frequent CT scanning and observation. Alternatively, the Indiana group recently reported 141 patients with normalization of STMs and radiographic disease <1 cm following chemotherapy for metastatic NSGCT.[32] With a median follow-up of 15 years, overall disease-specific survival was 97% and recurrence-free survival was 90%. Interestingly, 5% of patients in the IGCCCC good-risk classification recurred vs. 27% in intermediate or poor-risk categories (p = 0.001). Based on this data, it appears good- risk patients obtaining a complete response after primary chemotherapy can be safely observed without RPLND.

The rationale and benefits of post-chemotherapy surgery must be understood and balanced with the potential morbidity. The Indiana group first reported an overall complication rate of 21% and mortality rate of 0.8% following PC-RPLND. Defining major complications as any event requiring significant additional treatment and at least 2 more days of hospitalization, the group reported 106 of 144 complications as major (73.6%). Of the 106 complications, pulmonary events (31.9%), wound infection (20.1%) and small bowel obstruction (9.0%) were the most common. Although the majority of complications occurred in resections > 5 cm (93%), this study underscores the potential for morbidity in the post-chemotherapy setting.[33]

The most consistent long-term morbidity following PC- RPLND has been loss of antegrade ejaculation, caused by disruption of sympathetic nerve fibers along the sympathetic chain, post-sympathetic efferent fibers, or hypogastric plexus near the inferior mesenteric artery. For a PC-RPLND, virtually all experienced testicular cancer centers strongly recommend a full, bilateral RPLND with nerve-sparing, if technically feasible. With appropriate nerve-sparing, the dual goals of ejaculatory preservation and maximizing oncologic efficacy can be achieved during PC-RPLND, as Pettus *et al*. reported 136 men undergoing nerve-sparing PC-RPLND with 107 (79%) reporting antegrade ejaculation postoperatively and only 2 (1.5%) developing systemic relapse.[34] Predictors of ejaculatory dysfunction in this series were right-sided primary tumors as well as residual masses ≥ 5 cm. Minimizing the dissection boundaries to avoid nerve damage (using smaller, modified templates) unnecessarily increases the risk of unresected disease, which can threaten oncologic control. Carver *et al*. have estimated that if modified templates are used during PC-RPLND, rates of unresected cancer would range from 7-32%.[29] Investigators at Indiana University have performed modified PC-RPLND in a highly select group of 100 patients (^∼^10% of all their patients undergoing PC-RPLND) and noted four recurrences after modest follow-up, all occurring outside the boundaries of a full template dissection.[35]

## MANAGEMENT OF EXTRA-RETROPERITONEAL MASSES

Extra-retroperitoneal disease (ERP) or recurrence in NSGCT (CS ≥ III) is initially treated with chemotherapy and similar to post-chemotherapy RP disease, masses outside the abdomen need to be evaluated for surgical resection as they can harbor viable GCT or teratoma. The most common location of these recurrences is lung (^∼^70%). Horvath *et* *al*. reported on 15 patients over 10 years undergoing thoracotomy and reported an 80% complete response rate after wedge resection.[36] Pathologic findings at ERP resection are critical to prognosis, as Shayegan *et al*. found five-year disease-free survival in patients with fibrosis, teratoma, or viable GCT to be 92%, 53%, and 8%, respectively.[37] In cases with synchronous chest and RP masses, Steyerberg *et al*. showed PC-RPLND could be done first as both a staging and predictive model for chest disease, as fibrosis in the retroperitoneum predicted fibrosis in the lung 89% of the time.[38] Gels *et al*. looked at a similar group of patients and alternatively found in 20 patients with both RP and lung disease after resection, the pathology was discordant in 50% (10/20).[39] McGuire et al. also found 28% pathologic discordance for synchronous RP and chest lesions with 57% discordance for asynchronous lesions. Most concerning was 33% of synchronous and 39% asynchronous of chest lesions had worse pathology than the RP specimen.[40] The authors found no prognostic factors sufficiently accurate enough to preclude thoracotomy in this setting. As these procedures often require multiple surgical specialties, careful surgical planning is essential to determine optimal resection strategies.

A meta-analysis of patients with extra-RP disease by Carver cited an overall complication rate of 35% for simultaneous resection of multiple masses, comparable to those undergoing staged resections, supporting the safety of a simultaneous approach if time and operative factors permit.[41]

## CONCLUSIONS

The long-term prognosis of metastatic, good-risk NSGCT (CS IS-III) is excellent (92 – 94%) when appropriately integrating RPLND, cisplatin-based chemotherapy, and modern supportive care. Standard recommended therapies are based on stage, serum tumor marker status, and IGCCCG risk classification. Optimal outcomes are obtained with a multi-disciplinary collaborative approach to all metastatic NSGCT patients. Favorable outcomes are routinely expected but there remain several areas requiring further improvements: a better understanding of late relapses, chemoresistant GCT, and minimizing toxicity of treatment.
